# The Role of Resilience in Internet Addiction among Adolescents between Sexes: A Moderated Mediation Model

**DOI:** 10.3390/jcm7080222

**Published:** 2018-08-19

**Authors:** Cho Rong Nam, Da Heen Lee, Ji Yoon Lee, A Ruem Choi, Sun Ju Chung, Dai-Jin Kim, Soo-Young Bhang, Jun-Gun Kwon, Yong-Sil Kweon, Jung-Seok Choi

**Affiliations:** 1 Department of Psychiatry, SMG-SNU Boramae Medical Center, Seoul 07061, Korea; mmuseumm@gmail.com (C.R.N.); dhlee4370@gmail.com (D.H.L.); idiyuni91@gmail.com (J.Y.L.); choiar90@gmail.com (A.R.C.); sunjujung1991@gmail.com (S.J.C.); 2 Department of Psychiatry, Seoul St. Mary’s Hospital, College of Medicine, The Catholic University of Korea, Seoul 06591, Korea; kdj922@chol.com; 3 Department of Psychiatry, Eulji General Hospital, Seoul 01830, Korea; dresme@hanmail.net; 4 I Will Center, Seoul Metropolitan Boramae Youth Center, Seoul 07062, Korea; jun@boramyc.or.kr; 5 Department of Psychiatry, Uijeongbu St. Mary’s Hospital, The Catholic University of Korea College of Medicine, Seoul 06591, Korea; 6 Department of Psychiatry and Behavioral Science, Seoul National University College of Medicine, Seoul 03080, Korea

**Keywords:** internet addiction, resilience, behavioral inhibition/activation system, sex differences, moderated mediation

## Abstract

The behavioral inhibition/activation systems (BIS/BAS) have been considered to be predictors of Internet addiction, mediated by clinical variables such as anxiety and depression. However, resilience has been suggested as a protective factor toward Internet addiction, and certain sex differences in resilience buffering the effects of vulnerability have been reported. Thus, the aim of this study was to identify any role of resilience that might moderate the effects of BIS/BAS on Internet addiction through multiple clinical variables in boys and girls. A total of 519 middle-school students (268 boys and 251 girls, all 14 years old) were administered a questionnaire battery that measures Internet addiction, BIS/BAS, depression, anxiety, impulsivity, anger, and resilience. We used the PROCESS macro in SPSS to perform moderation and mediation analysis. Findings revealed that although a somewhat similar mediation model was supported in both sexes, moderating effects of resilience only emerged in girls. The results showed a protective role of resilience differing between sexes. These results suggest that clinicians should consider sex in the way resilience works as a protective factor against Internet addiction and focus on mitigating the effects of vulnerability by enhancing resilience in female Internet addicts.

## 1. Introduction

The notion that some rewarding non-substance-related behaviors can drive a person to compulsively engage in such behaviors and cause negative consequences has arisen since the concept of behavioral addiction was established by pathological gambling in the Diagnostic and Statistical Manual of Mental Disorders, 4th Edition (DSM-IV) [1]. Excessive Internet usage creates psychological, social, school, and/or work difficulties in a person’s life. Specifically, it may cause mental health problems such as anxiety, depression, and impulsivity, as well as isolation from social environment [2], leading to academic failure or family problems [3]. In line with such circumstances, Internet Gaming Disorder was included as a condition for further study in DSM-V [4]. Due to its considerable influence and high accessibility, the Internet has been considered a hot topic in behavioral addiction [5,6,7,8]. In a bid to understand this new area of addiction, several relevant factors such as temperament or comorbidity have been investigated [9,10,11].

To begin with, Gray’s [12,13] neuropsychological theory is known as an effective view for understanding and explaining basic human behaviors, especially regarding addiction. Gray suggested that two neural mechanisms are in charge of controlling emotions and behavior: an aversion system and an appetitive system. These are also called the behavioral inhibition system (BIS) and the behavioral approach system (BAS), respectively. Facing certain stimuli in an environment, one of these systems is activated. BIS is associated with stimuli conditioned for punishment or the termination of rewards, whereas BAS is associated with stimuli relevant to rewards or the termination of punishment [14,15]. According to these systems, a person’s personality is based on sensitivity toward stimuli associated with negative/positive reinforcement, that is, BIS/BAS, and individual differences in personality arise as a result of variation in such sensitivity [16,17].

In accordance with this notion, several studies have investigated relationships between BIS/BAS and addiction. Despite inconsistency in detail, high levels of BAS have been found to predict pathological engagement in compulsive and approach behaviors including substance or alcohol abuse [15,18,19]. In some cases, low BIS aggravated the influence of high BAS [20]. Similarly, high BAS and low BIS have been suggested to be a predictor of alcohol or drug abuse [21,22,23] and pathological gambling [24]. On the other hand, unlike the apparent role of BAS, the effect of BIS on addiction seems complicated. Few studies found that low BIS predicted drug use [22,24], yet another study found no relationship between BIS and alcohol abuse-related variables [25]. In terms of Internet addiction, both BIS and BAS, with high scores, seem to play important roles [26,27,28,29]. It can be inferred from these conflicting results that Internet addiction has a distinctive mechanism [30], differing from other addictions regarding the role of BIS.

Furthermore, these inconsistencies may indicate that BIS/BAS itself cannot sufficiently account for the mechanism of addiction. Thus, there should be some bridge connecting the two spots: BIS/BAS as departure points and addiction as an arrival point. Davidson [31] suggested that the difference in sensitivity toward BIS/BAS makes a difference in affective style. BIS is related to neuroticism [32], which is closely related with negative affectivity and emotional instability [33]. In contrast, BAS is considered to be a predisposition to impulsivity [13]. Given that highly impulsive or anxious individuals engage in addictive behaviors to deal with emotional distress [34], these clinical variables may be the bridge that fills the space between BIS/BAS and addiction. Previous studies have suggested the predictive role of clinical variables, including anxiety and impulsivity as well as depression and anger, on Internet addiction [25,35,36,37]. Furthermore, in a recent study that was conducted with Korean high school students, impulsivity, anxiety, and depression mediated the positive relationship between BIS/BAS and Internet addiction [28]. Therefore, it is plausible to hypothesize that BIS/BAS affect one of the pathways to Internet addiction as vulnerability factors, mediated by clinical variables such as depression, anxiety, impulsivity, and anger.

Although BIS/BAS acts as risk factor, resilience has been suggested as a protective factor against various psychopathologies, including addiction [38,39,40]. Resilience is a broad variable, defined as “the personal qualities that enable one to thrive in the face of adversity” [41], and considered to be the availability of constructive coping strategies. That is, a resilient individual can deal with stress and successfully reduce negative psychological outcomes, such as anxiety and depression [42], and the effects of vulnerability can be buffered by high levels of resilience [43]. Such protective effect of resilience has been supported in Internet addiction, in the same manner. Resilience decreased negative psychological effects that are commonly accompanied by Internet addiction [38]. Moreover, even in teens already showing high levels of Internet addiction, resilience protected them from engaging in risky behaviors online [44]. Having more internal resources to cope with stress and a positive view toward adverse situations, resilient adolescents indulged less in Internet use to control distressful emotions [45].

However, it has been proposed that there are sex differences in the pathway described above. Firstly, some studies reported slightly higher BIS in women than in men and complex results of BAS depending on subscales, whereas others showed no significant differences [21,25,46,47,48,49]. Regarding clinical variables, Internet addiction was mainly correlated with impulsivity or aggression in men, whereas correlations with depression or anxiety were prominent in women [37,50,51]. Furthermore, there seems to be a clear distinction with respect to resilience-related factors between sexes [52,53]. In general, girls tended to show higher scores in resilience [54,55] along with constructive coping strategies, such as seeking social support and problem solving, whereas boys showed higher scores in avoidant coping [56,57,58]. Moreover, only in girls, resilience had a negative association with the possibility of Internet addiction [37] and moderated the relationship between depression and Internet addiction [50].

**Figure 1:**
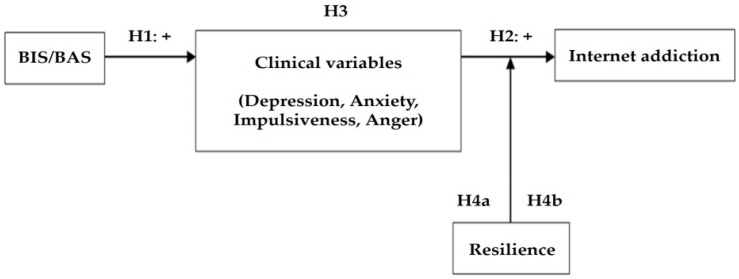
Hypothesized conceptual model. Note: BIS/BAS = Behavioral Inhibition System/Behavioral Activation System; H1 = hypothesis 1; H2 = hypothesis 2; H3 = hypothesis 3; and H4 = hypothesis 4.

With this background, this study aimed to identify a pathway by which resilience moderated the effects of BIS/BAS on Internet addiction through multiple clinical variables in adolescents. Further, we considered that there would be sex differences in this pathway, both in the mediation model and moderation model, and thus examined it separately for boys and girls. To our knowledge, the comprehensive studies incorporating risk factors, mediating factors, protective factors, and sex differences in Internet addiction are scarce. Thus, we expected that this study would provide an in-depth view of Internet addiction in adolescents. Our hypotheses are as follows: (1) BIS/BAS will be positively correlated with clinical variables, (2) clinical variables will be positively correlated with Internet addiction, (3) clinical variables will mediate the relationship between BIS/BAS and Internet addiction, (4a) the positive relationship between clinical variables and Internet addiction will be stronger for adolescents with low resilience than for those with high resilience, (4b) resilience will moderate the indirect effect of BIS/BAS on Internet addiction (through clinical variables). Specifically, clinical variables will mediate the indirect effect of BIS/BAS when resilience is low but not when high, and (5) the pathway through which resilience moderates the effects of BIS/BAS on Internet addiction, through multiple clinical variables, will differ by sex. Figure 1 depicts overall hypotheses of this study.

## 2. Methods

### 2.1. Participants

Participants were recruited from after-school programs that were held at some middle schools located in Seoul, Korea. All students received an explanation about the research and we asked for informed consent from themselves as well as their parents prior to participation. In total, 519 middle-school students in Seoul, Korea were administered a questionnaire battery (boys = 268, girls = 251; all the same age of 14 years old). Participants received an explanation about the research and completed the self-administered questionnaire at school. Gift certificates were provided as a reward for participation.

### 2.2. Measures

All questionnaires we used have been validated in Korean [59,60,61,62,63,64,65].

#### 2.2.1. Young’s Internet Addiction Test (Y-IAT)

The Internet addiction scale developed by Young [66] is rated on a five-point scale. Total scores were calculated according to Young’s [66] method, with possible scores for all 20 items ranging from 20 to 100. Cronbach’s α coefficient of Korean version was 0.91 [60].

#### 2.2.2. Behavioral Inhibition System/Behavioral Activation System Scales (BIS/BAS)

We used BIS/BAS scales [14] to assess sensitivity to rewards and punishments. These scales consist of 20 items rated on a four-point Likert scale, with 7 items for BIS and 13 items for BAS. The BAS scale can be subdivided into three subscales: fun-seeking, reward responsiveness, and drive. Cronbach’s α coefficient for all subscale was above 0.78 in Korean version [62].

#### 2.2.3. Beck Depression Inventory (BDI-II)

The BDI-II [67] is a 21-item self-reported questionnaire in which each item consists of four statements indicating different levels of severity of a particular symptom experienced during the past week. Cronbach’s α coefficient of Korean version was 0.88 [64].

#### 2.2.4. Beck Anxiety Inventory (BAI)

The BAI [68] consists of 21 symptoms that are rated on a four-point scale measuring the severity of certain symptoms experienced during the past week. Cronbach’s α coefficient of Korean version was 0.94 [65].

#### 2.2.5. Barratt Impulsiveness Scale, Version 11 (BIS-11)

BIS-11 [69] assesses impulsivity and includes three subscales: cognitive impulsiveness, motor impulsiveness, and non-planning impulsiveness. Cronbach’s α coefficient of Korean version was 0.80 [63].

#### 2.2.6. State-Trait Anger Expression Inventory (STAXI)

Anger was assessed using the Korean version of State-Trait Anger Expression Inventory (K-STAXI) [59]. This scale assesses how often the respondent experiences each of 10 anger-related feelings. Cronbach’s α coefficient was 0.87.

#### 2.2.7. Connor-Davison Resilience Scale (CDRS)

The CDRS contains 25 items that measure resiliency on a five-point Likert scale [41]. Total scores range from 0 to 100, in which higher scores indicate greater resilience. Cronbach’s α coefficient of Korean version was 0.93 [61].

### 2.3. Statistical Analyses

We tested our study hypotheses in two interlinked steps. Firstly, we tested simple mediation models for each clinical variable (Hypotheses 1–3). Next, we integrated the proposed moderator variable into the models (Hypothesis 4a) and examined the overall moderated mediation empirically (Hypothesis 4b). These steps were conducted separately for each sex (Hypothesis 5). SPSS software (v. 21 for Windows, IBM Corp, Armonk, NY, USA) was used for statistical analyses. A *p*-value < 0.05 was considered to indicate statistical significance. Prior to the analysis, all continuous measures were mean-centered to avoid multicollinearity problems caused by correlations among variables [70,71].

#### 2.3.1. Test of Mediation

Hypotheses 1, 2, and 3 collectively suggest indirect effect models, in which the relationships between BIS/BAS and Internet addiction are transmitted through clinical variables. We followed the procedures of Baron and Kenny [72] to examine such mediation hypotheses, conducting regression analyses with variables as follows: Y-IAT as a criterion variable, BIS/BAS as predictors separately, and clinical variables as mediators separately. Notably, there has been an updated account of this procedure [73]. According to this updated mediation procedure, the necessity of the significant direct effect of initial, independent variable X to outcome Y is no longer essential. Therefore, the main effect may be weak or nonsignificant and an indirect effect may exist [73,74]. For this reason, we tested the mediation hypotheses (Hypotheses 1–3) using an application provided by Hayes [75] and executed by Cole et al. [76] and Chen et al. [77]. The SPSS macro called PROCESS is a computational tool for path analysis-based moderation and mediation analysis as well as for their combination (*conditional process model*) [75,78]. PROCESS can facilitate estimations of the indirect effect by using the SOBEL test and a bootstrap approach to obtain the confidence interval (CI) and to incorporate the stepwise procedure suggested by Baron and Kenny [72].

#### 2.3.2. Test of Moderated Mediation

Hypothesis 4a predicted that resilience would moderate positive relationships between clinical variables and Internet addiction. Assuming that this can be proven, the strength of the hypothesized indirect (mediation) effect is plausibly conditional on the value of the moderator of resilience, as described in Hypothesis 4b. Such effect is called the “conditional indirect effect” or “moderated mediation” [79]. We also used PROCESS [75] to examine Hypotheses 4a and 4b. PROCESS easily executes bootstrapping methods and provides a method for probing the significance of conditional indirect effects at different moderator variable values.

### 2.4. Ethics

All subjects received an explanation about the research and provided written informed consent prior to participation. The study was approved by Institutional Review Board of Seoul St. Mary’s Hospital, Seoul, Republic of Korea (KC13ONSI0080, 8 April 2013) and was conducted in accordance with the Declaration of Helsinki.

## 3. Results

### 3.1. Correlations Between Overall Variables

Table 1 presents the descriptive statistics and correlations for overall variables. Y-IAT was correlated significantly with all of the variables, but only CDRS showed a negative correlation (*r* = −0.122, *p* < 0.01). These results can be interpreted as basic evidence that resilience is a protective factor against Internet addiction, because the higher the resilience, the lower the Internet addiction score. BIS/BAS was correlated positively with most clinical variables, but BIS-11 showed no significant correlation with BIS (*r* = 0.080, *p* > 0.05) or the BAS-drive subscale (*r* = 0.050, *p* > 0.05).

### 3.2. Sex Differences in Overall Variables

Next, we examined sex differences using *t*-tests (Table 2). The difference in Y-IAT was prominent, with a higher score in boys than girls (*t* = 5.723, *p* < 0.001, *Cohen’s d* = 0.50). BIS and BAS-reward responsiveness were higher in girls than boys (*t* = −4.804, *p* < 0.001, *Cohen’s d* = −0.43; *t* = −1.984, *p* < 0.05, *Cohen’s d* = −0.18), as were BDI (*t* = −5.085, p < 0.001, *Cohen’s d* = −0.45) and STAXI (*t* = −2.851, *p* < 0.01, *Cohen’s d* = −0.25). CDRS did not differ significantly between sexes (*t* = 0.612, *p* > 0.05).

### 3.3. Tests of Mediation

#### 3.3.1. Boys

**Figure 2:**
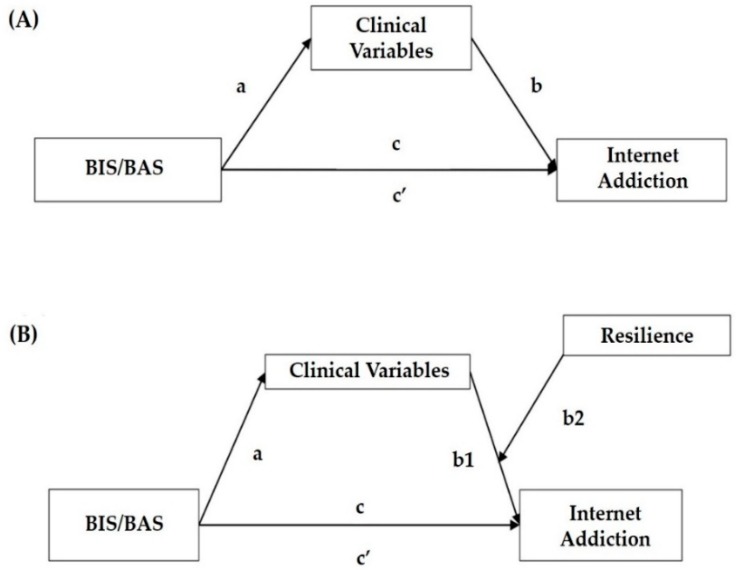
(**A**) Simple mediation and (**B**) moderated mediation model. Note. BIS/BAS = Behavioral Inhibition System/Behavioral Activation System; a, b, c, and c’ represent each path coefficient; a: Effect of BIS/BAS on mediating variable; b: Effect of mediating variables on Y-IAT’; c: Direct effect of BIS/BAS on Y-IAT with controlling mediating effect; and c’: Total effect of BIS/BAS without controlling mediating effect.

Figure 2 and Table 3 present the results regarding Hypotheses 1–3 in boys. BIS, BAS-reward responsiveness, and BAS-drive were associated positively with BDI, BAI, and STAXI, as indicated by the significant non-standardized regression coefficients. Moreover, BAS-fun seeking was positively associated with all clinical variables. Thus, Hypothesis 1 was supported (see column a of Table 3). Furthermore, positive relationships between the clinical variables mentioned above and Y-IAT, controlling for BIS/BAS, were found, supporting Hypothesis 2 (see column b of Table 3). Finally, the positive relationship of BIS/BAS and Y-IAT was found to be mediated by clinical variables, as hypothesized (see columns c and c’ of Table 3). More specifically, BIS was fully mediated by BDI, BAI, and STAXI. BAS-reward responsiveness and BAS-drive were partially mediated by BDI and BAI and fully mediated by STAXI. BAS-fun seeking was partially mediated by all clinical variables. In other words, BIS/BAS were found to have positive indirect effects on Y-IAT through clinical variables and formal two-tailed significance tests confirmed that these indirect effects were significant (see Indirect effect column (ab) of Table 3). Thus, these regression results for simple mediation revealed that Hypotheses 1, 2, and 3 were at least partially supported.

#### 3.3.2. Girls

Figure 2 and Table 4 present the results regarding Hypotheses 1–3 in girls, showing mostly similar but somewhat different results than those of boys. Firstly, BIS was associated positively with BDI, BAI, and STAXI, as indicated by the significant non-standardized regression coefficients. BAS-reward responsiveness and BAS-fun seeking were associated positively with all clinical variables. BAS-drive showed positive associations with BAI and STAXI. Thus, Hypothesis 1 was supported (see column a of Table 4). Also, positive relationships between the clinical variables mentioned above and Y-IAT, controlling for BIS/BAS, were found, supporting Hypothesis 2 (see column b of Table 4). Finally, the positive relationship of BIS/BAS and Y-IAT was found to be mediated by clinical variables, as hypothesized. (see columns c and c’ of Table 4). More specifically, BIS was fully mediated by BDI, BAI, and STAXI. BAS-reward responsiveness was partially mediated by BDI, BAI, BIS-11, and STAXI. BAS-drive was partially mediated by BAI and STAXI. BAS-fun seeking was partially mediated by all clinical variables. In other words, BIS/BAS were found to have positive indirect effects on Y-IAT through clinical variables and formal two-tailed significance tests confirmed that these indirect effects were significant (see Indirect effect column (ab) of Table 4). Thus, these regression results for simple mediation revealed that Hypotheses 1, 2, and 3 were at least partially supported.

### 3.4. Tests of Moderated Mediation

#### 3.4.1. Boys

Although the simple mediation model was confirmed through supporting results for Hypotheses 1–3, no cross-product term between clinical variables and CDRS on Y-IAT reached statistical significance (BDI: B = 0.002, *t* = 0.321, *p* = 0.748 for BIS; B = 0.002, *t* = 0.329, *p* = 0.743 for BAS_r; B = 0.001 *t* = 0.257, *p* = 0.797 for BAS_d; B = −0.001, *t* = −0.049, *p* = 0.961 for BAS_f; BAI: B = 0.007, *t* = 1.457, *p* = 0.146 for BIS; B = 0.007, *t* = 1.546, *p* = 0.123 for BAS_r; B = 0.007, *t* = 1.411, *p* = 0.160 for BAS_d; B = 0.006, *t* = 1.311, *p* = 0.191 for BAS_f; BIS-11: B = −0.007 *t* = −1.418, *p* = 0.157 for BAS_f). Thus, Hypotheses 4a and 4b were rejected in boys.

#### 3.4.2. Girls

Figure 2 and Table 5 show the results related to Hypotheses 4a and 4b in girls. In Hypothesis 4a, we suggested that positive relationships between clinical variables and Internet addiction would be more robust for adolescents with low resilience than for those with higher resilience. The results indicated that the cross-product terms between BAI and CDRS on Y-IAT were significant for all of the personality variables in girls (B = −0.008, *t* = −2.480, *p* < 0.05 for BIS; B = −0.008, *t* = −2.637, *p* < 0.01 for BAS_r; B = −0.007 *t* = −2.398, *p* < 0.05 for BAS_d; B = −0.007, *t* = −2.284, *p* < 0.05 for BAS_f). The cross-product terms between BIS-11 and CDRS on Y-IAT were significant for BAS-reward responsiveness and BAS-fun seeking (see column b2 of Table 5).

**Figure 3:**
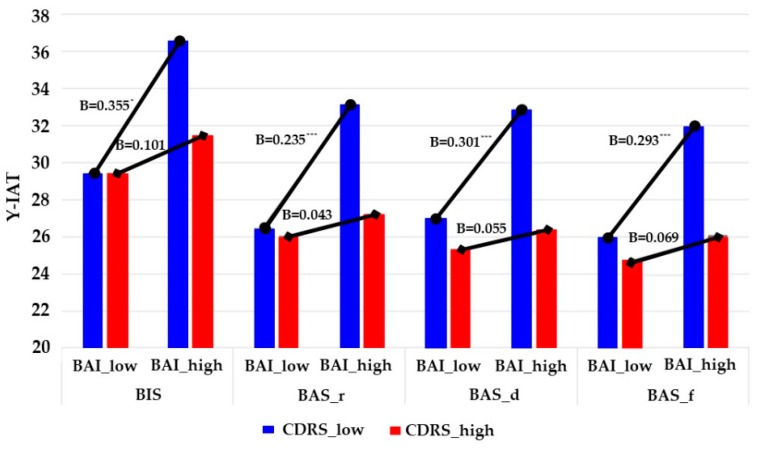
Internet addiction predicted by BIS/BAS through anxiety at different values of resilience in female students. Note: *N* = 251. Unstandardized regression coefficients are reported. BIS = Behavioral Inhibition System scale; BAS_r = Behavioral Activation System scale_Reward responsiveness; BAS_d = Behavioral Activation System scale_Drive; BAS_f = Behavioral Activation System scale_Fun seeking; BAI = Beck Anxiety Inventory; Y-IAT = Young’s Internet Addiction Test; low = one standard deviation below mean; high = one standard deviation above mean. * *p* < 0.05, *** *p* < 0.001.

**Figure 4:**
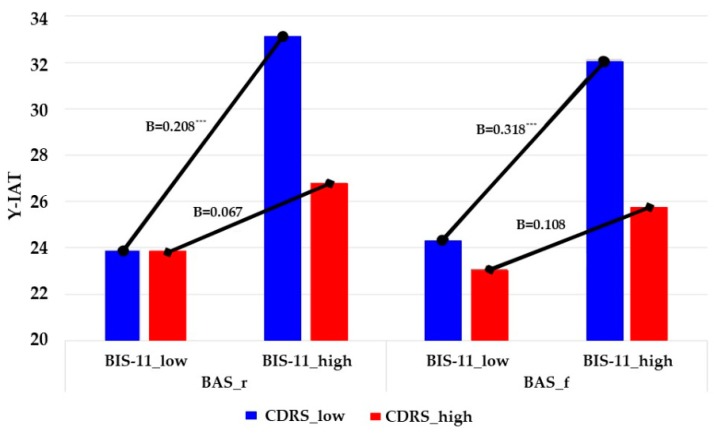
Internet addiction predicted by BIS/BAS through impulsivity at different values of resilience in female students. Note: *N* = 251. Unstandardized regression coefficients are reported. BAS_r = Behavioral Activation System scale_Reward responsiveness; BAS_f = Behavioral Activation System scale_Fun seeking; BIS-11 = Barratt Impulsiveness Scale; CDRS = Connor-Davidson Resilience Scale; Y-IAT = Young’s Internet Addiction Test; low = 1 standard deviation below mean; high = 1 standard deviation above mean. *** *p* < 0.001.

Furthermore, we plotted these cross-product terms, developing separate equations that used one standard deviation above and below the mean of CDRS to represent high versus low for each respective variable [70]. Figure 3 and Figure 4 show the interactions between BAI/BIS-11 and CDRS. We also performed simple slope analyses, following the process described by Preacher et al. [79]. Consistent with Hypothesis 4a, the slope of the relationship between BAI and Y-IAT was steep for students with low CDRS (simple slope = 0.390, *t* = 2.230, *p* = 0.027 for BIS; simple slope = 0.353, *t* = 9.067, *p* = 0.000 for BAS_r; simple slope = 0.320, *t* = 8.040, *p* = 0.000 for BAS_d; simple slope = 0.323, *t* = 8.730, *p* = 0.000 for BAS_f), whereas the slope was relatively shallow for those with higher CDRS (simple slope = 0.111, *t* = 0.487, *p* = 0.626 for BIS; simple slope = 0.064, *t* = 0.436, *p* = 0.663 for BAS_r; simple slope = 0.058, *t* = 0.393, *p* = 0.694 for BAS_d; simple slope = 0.076, *t* = 0.520, *p* = 0.603 for BAS_f). Similarly, the slope of the relationship between BIS-11 and Y-IAT was steep for students with low CDRS (simple slope = 0.509, *t* = 42.771, *p* = 0.000 for BAS_r; simple slope = 0.430, *t* = 29.292, *p* = 0.000 for BAS_f), whereas the slope was relatively shallow for those with higher CDRS (simple slope = 0.162, *t* = 1.064, *p* = 0.288 for BAS_r; simple slope = 0.146, *t* = 0.962, *p* = 0.337 for BAS_f).

Along with these results, the conditional indirect effects of BIS/BAS on Y-IAT through BAI/BIS-11 also supported our hypothesis (Conditional indirect at different values of the moderator in column ab1 of Table 5). Regarding BAI, normal-theory tests suggested that one of the three conditional indirect effects—based on moderator values at one standard deviation below the mean—was positive and significantly different from zero. Bootstrap CIs corroborated these results. That is, indirect and positive effects of BIS/BAS on Y-IAT through BAI were observed when CDRS was low, but not when CDRS was moderate to high. This result indicates that moderate to high levels of resilience attenuated the influence of BIS/BAS on Internet addiction, which was transmitted by anxiety. Regarding BIS-11, two of the three conditional indirect effects—based on moderator values at the mean and at one standard deviation below the mean—were positive and significantly different from zero. Bootstrap CIs also corroborated these results. That is, indirect and positive effects of BIS/BAS on Y-IAT through BIS-11 were observed when CDRS was low to moderate, but not when CDRS was high. It implies that high levels of resilience lessened the effects of BIS/BAS on Internet addiction, which was transmitted by impulsivity. Thus, Hypothesis 4b was supported in girls. Hypothesis 5 was supported accordingly, owing to Hypotheses 4a and 4b having been rejected in boys.

## 4. Discussion

In this study, we sought to identify sex-specific pathways where resilience moderated the indirect effect of BIS/BAS on Internet addiction via depression, anxiety, impulsivity, and anger.

To begin with, as a departure point of the overall model, sex differences in BIS were consistent with previous studies, with higher BIS and BAS_r scores in girls than boys [21,25], whereas BAS-drive and BAS-fun seeking showed no significant sex difference. Regarding mediating variables, girls seemed to score higher in depression and anger than boys. This result seems to be in line with the previous studies that found girls tend to suppress anger at a higher rate than boys [80] and anger-inward was significantly more highly correlated with depression among girls than boys [81]. However, mediation models turned out to be similar between sexes except for minor differences. In both sexes, depression and anxiety along with anger showed significant mediating effects in the positive relationship between BIS/BAS and Internet addiction. Impulsivity mediated the effects of BAS-fun seeking on Internet addiction in both sexes as well, but acted as a mediator in the pathway of BAS-reward responsiveness affects internet addiction only in girls. Finally, as an arrival point of the model, Internet addiction score was markedly higher in boys (*Cohen’s d* = 0.50), which is in concordance with previous studies [37,50,51]. Interestingly, although there was no significant sex difference in resilience, the buffering effect of resilience toward Internet addiction only emerged in girls. This female-specific protective effect of resilience could be inferred from previous studies [52,53,56]. However, the fact that resilience itself did not differ by sex seems inconsistent with previous studies [54,55] and leaves room for comprehending the role of resilience in Internet addiction. Considering resilience is a developmental concept that can be fostered through positive interactions with the environment [82], adolescents included in this study might be too young to show some sex differences in the resilience score. In fact, previous studies that suggested higher resilience in women had much older participants than our study [54,55].

Then, what makes girls benefit more from resilience than boys, despite having no significant difference in the level of resilience? The answer seems to lie within the distinctive characteristics of Internet use according to sex. When facing stress, girls are more likely to seek out and receive support than boys [53]. Likewise, girls tend to deal with daily stressful events using social aspects, whereas boys are more likely drawn to physical recreation [52]. This tendency seems to carry over into the online dimension, with girls spending more time on the Internet trying to interact with others via Messenger or social networking services, whereas boys play online games [37,83]. Everall et al. [84] found that involvement in diverse interpersonal relationships and extracurricular activities is relevant to resilience. Because girls pursue social connections both online and offline, urges to communicate could be satisfied through different channels other than the Internet among those who are resilient. Although social support provided in online space could be helpful to a certain degree, it is relatively temporary and unstable compared to what is derived from offline or real life interactions [85]. Therefore, girls with low resilience might cling to online relationships for the sake of social connection, only to experience more negative emotions, such as depression or anxiety, due to insufficient support and this vicious circle then continues. In contrast, boys might not significantly differ in using the Internet by level of resilience, because they simply try to have fun in online space [86]. In other words, those findings from previous studies enabled us to carefully assume that even if girls do not have higher resilience scores than boys, they could still benefit more from resilience than boys owing to their motives for using the Internet, characterized by seeking social connection. Moreover, the relationship between resilience and positive internal resources was found to be stronger in women than in men [87]. Therefore, resilience can buffer the risk of Internet addiction only in girls.

This study has several limitations. Above all, we analyzed each model including one mediator at a time, rather than including all four mediators in a single model. Of course, including various mediators that have correlations among themselves in a multiple mediation model could differentiate a causal relationship from spurious or subsidiary relationships. However, it risks causing a multicollinearity problem and lowering statistical power [88]. Considering that the clinical variables in this study had correlations among themselves, we chose to have a higher statistical threshold by adopting simple mediation models. Secondly, there were few samples with clinically high scores (>70) on Y-IAT. Most participants were within the range of ‘healthy’ Internet users, with 75.3% of samples scoring below 40. Thus, extending the findings of this study should be preceded by further studies including more severe samples. Thirdly, we relied solely on subjective measures via self-reported scales; future studies should investigate the protective role of resilience more thoroughly by encompassing more objective measures, such as reports from acquaintances. Finally, there could be the limits of representativeness due to the small sample size and restricted age range. Although we recruited adolescents aged 14 years old that were known to be more susceptible to developing Internet addiction [89], it is necessary to be cautious when generalizing these results.

Despite these limitations, we showed a protective role of resilience differing between sexes by testing a moderated mediation model with results supporting five hypotheses. Based on the current results, clinicians should consider focusing on sex-specific intervention for increasing resilience among girls to prevent Internet addiction. Furthermore, we recommend examining more thoroughly the concept of ‘seeking social support online’, which has been suggested as a key difference in sex regarding motives for using the Internet, in the testing model to investigate the process by which resilience lessens the risk of Internet addiction in girls.

## Figures and Tables

**Table 1:** Mean, standard deviation, and correlation of overall variables.

	1	2	3	4	5	6	7	8	9	10
1. BIS										
2. BAS_r	0.581 **									
3. BAS_d	0.413 **	0.769 **								
4. BAS_f	0.375 **	0.721 **	0.714 **							
5. BDI	0.389 **	0.176 **	0.145 **	0.182 **						
6. BAI	0.377 **	0.264 **	0.280 **	0.284 **	0.674 **					
7. BIS-11	0.080	0.124 **	0.050	0.173 **	0.297 **	0.322 **				
8. STAXI	0.416 **	0.446 **	0.410 **	0.388 **	0.295 **	0.310 **	0.169 **			
9. CDRS	−0.122 **	0.118 **	0.196 **	0.132 **	−0.304 **	−0.215 **	−0.330 **	−0.019		
10. Y-IAT	0.117 **	0.199 **	0.232 **	0.273 **	0.197 **	0.333 **	0.321 **	0.243 **	-0.122 **	
M	17.42	11.85	8.58	8.38	7.54	6.55	63.19	54.04	62.55	33.84
SD	3.691	3.716	2.889	2.909	7.867	8.36	8.608	10.68	18.13	12.696

** *p* < 0.01. Note: N = 519. BIS = Behavioral Inhibition System scale; BAS_r = Behavioral Activation System scale_Reward responsiveness; BAS_d = Behavioral Activation System scale_Drive; BAS_f = Behavioral Activation System scale_Fun seeking; BDI = Beck Depression Inventory; BAI = Beck Anxiety Inventory; BIS-11 = Barratt Impulsiveness Scale; STAXI = State-Trait Anger Expression Inventory; CDRS = Connor-Davidson Resilience Scale; Y-IAT = Young’s Internet Addiction Test.

**Table 2:** Sex differences in overall variables.

	Mean (SD)		*t*(*p*)
	Boys	Girls	
1. BIS	16.68 (3.344)	18.22 (3.881)	−4.804 (0.000) ***
2. BAS_r	11.53 (3.845)	12.18 (3.551)	−1.984 (0.047) *
3. BAS_d	8.51 (3.040)	8.65 (2.724)	−0.546 (0.585)
4. BAS_f	8.33 (3.071)	8.44 (2.730)	−0.417 (0.677)
5. BDI	5.87 (6.444)	9.33 (8.812)	−5.085 (0.000) ***
6. BAI	5.92 (7.310)	7.24 (9.319)	−1.783 (0.075)
7. BIS-11	63.25 (8.161)	63.12 (9.078)	0.162 (0.872)
8. STAXI	52.75 (11.393)	55.41 (9.699)	−2.851 (0.005) **
9. CDRS	63.02 (19.025)	62.05 (17.148)	0.612 (0.541)
10. Y-IAT	36.83 (13.045)	30.66 (11.510)	5.723 (0.000) ***

* *p* < 0.05, ** *p* < 0.01, *** *p* < 0.001. Note: No. for boys = 268; No. for birls = 251. BIS = Behavioral Inhibition System scale; BAS_r = Behavioral Activation System scale_Reward responsiveness; BAS_d = Behavioral Activation System scale_Drive; BAS_f = Behavioral Activation System scale_Fun seeking; BDI = Beck Depression Inventory; BAI = Beck Anxiety Inventory; BIS-11 = Barratt Impulsiveness Scale; STAXI = State-Trait Anger Expression Inventory; CDRS = Connor-Davidson Resilience Scale; Y-IAT = Young’s Internet Addiction Test.

**Table 3:** Regression results for simple mediation in boys.

Personality Features	Clinical Features	B				
		a	b	c	c’	Indirect Effect (ab)
1. BIS	BDI	0.614 ***	0.608 ***	0.300	0.673 **	0.373 ***
	BAI	0.785 ***	0.629 ***	0.180		0.494 ***
	STAXI	1.335***	0.323 ***	0.242		0.431 ***
2. BAS_r	BDI	0.230 *	0.607 ***	0.622 **	0.761 ***	0.140 ***
	BAI	0.519 ***	0.593 ***	0.454 *		0.308 ***
	STAXI	1.500***	0.297 ***	0.316		0.445 ***
3. BAS_d	BDI	0.368 *	0.587 ***	0.859 ***	1.075 ***	0.216 ***
	BAI	0.703 ***	0.577 ***	0.669 **		0.406 ***
	STAXI	1.853***	0.277 ***	0.563		0.512 ***
4. BAS_f	BDI	0.372 **	0.574 ***	0.992 ***	1.205 ***	0.214 *
	BAI	0.742 ***	0.554 ***	0.794 **		0.411 ***
	BIS-11	0.347 *	0.488 ***	1.036 ***		0.169 *
	STAXI	1.694***	0.256 ***	0.772 **		0.433 ***

* p < 0.05, ** *p* < 0.01, *** *p* < 0.001. Note: N = 268. Unstandardized regression coefficients are reported. Bootstrap resample size = 5000. B refers to regression coefficient of each path, designated by (a), (b), (c), (c’) and (ab) in the table and figure. BIS = Behavioral Inhibition System scale; BAS_r = Behavioral Activation System scale_Reward responsiveness; BAS_d = Behavioral Activation System scale_Drive; BAS_f = Behavioral Activation System scale_Fun seeking; BDI = Beck Depression Inventory; BAI = Beck Anxiety Inventory; BIS-11 = Barratt Impulsiveness Scale; and STAXI = State-Trait Anger Expression Inventory.

**Table 4:** Regression results for simple mediation in girls.

Personality Features	Clinical Features	B				
		a	b	c	c’	Indirect Effect (ab)
1. BIS	BDI	0.884 ***	0.234 **	0.338	0.545 **	0.207 *
	BAI	0.908 ***	0.435 ***	0.149		0.396 ***
	STAXI	1.057 ***	0.251 **	0.280		0.265 ***
2. BAS_r	BDI	0.468 **	0.244 **	0.638 **	0.752 ***	0.114 *
	BAI	0.663 ***	0.413 ***	0.478 *		0.274 **
	BIS-11	0.408 *	0.375 ***	0.599 **		0.153 *
	STAXI	0.963 ***	0.229 **	0.531 *		0.221 **
3. BAS_d	BAI	0.940 ***	0.409 ***	0.623 *	1.007 ***	0.384 ***
	STAXI	1.045 ***	0.235 **	0.761 **		0.246 **
4. BAS_f	BDI	0.630 **	0.227 **	1.079 ***	1.222 ***	0.143 *
	BAI	0.905 ***	0.392 ***	0.867 ***		0.354 ***
	BIS-11	0.737 ***	0.965 ***	0.965 ***		0.257 **
	STAXI	1.045 ***	0.215 **	0.997 ***		0.225 *

*p* < 0.05, ** *p* < 0.01, *** *p* < 0.001. Note: N = 251. Unstandardized regression coefficients are reported. Bootstrap resample size = 5000. B refers to regression coefficient of each path, designated by (a), (b), (c), (c’) and (ab) in the table and figure. BIS = Behavioral Inhibition System scale; BAS_r = Behavioral Activation System scale_Reward responsiveness; BAS_d = Behavioral Activation System scale_Drive; BAS_f = Behavioral Activation System scale_Fun seeking; BDI = Beck Depression Inventory; BAI = Beck Anxiety Inventory; BIS-11 = Barratt Impulsiveness Scale; and STAXI = State-Trait Anger Expression Inventory.

**Table 5:** Regression results for moderated mediation model in girls.

Personality Feature	Clinical Feature	B			Conditional Indirect Effect (ab1) at Different Values of the Moderator				
		a	b1	b2		Boot Indirect Effect	Boot SE	95% Boot LLCI	95% Boot ULCI
1. BIS	BAI	0.908 ***	0.251 ***	−0.008 *	−1SD	0.355 *	0.145	0.111	0.698
					M	0.228	0.134	−0.056	0.491
					+1SD	0.101	0.224	−0.406	0.475
2. BAS_r	BAI	0.663 ***	0.209 *	−0.008 **	−1SD	0.235 ***	0.122	0.049	0.559
					M	0.139	0.097	−0.035	0.344
					+1SD	0.043	0.148	−0.312	0.272
	BIS-11	0.408 *	0.336 ***	−0.010 *	−1SD	0.208 ***	0.096	0.061	0.445
					M	0.137	0.065	0.040	0.299
					+1SD	0.067	0.053	−0.008	0.211
3. BAS_d	BAI	0.940 ***	0.189 *	−0.008*	−1SD	0.301 ***	0.171	0.053	0.757
					M	0.178	0.140	−0.070	0.476
					+1SD	0.055	0.205	−0.430	0.381
4. BAS_f	BAI	0.905 ***	0.199 *	−0.007 *	−1SD	0.293 ***	0.161	0.048	0.693
					M	0.181	0.123	−0.048	0.448
					+1SD	0.069	0.192	−0.486	0.342
	BIS-11	0.737 ***	0.289 ***	−0.008 *	−1SD	0.318 ***	0.126	0.118	0.611
					M	0.213	0.089	0.072	0.422
					+1SD	0.108	0.082	−0.027	0.304

* p < 0.05, ** *p* < 0.01, *** *p* < 0.001. Note: N = 251. Unstandardized regression coefficients are reported. Bootstrap resample size = 5000. B refers to regression coefficient of each path, designated by (a), (b), (c), (c’) and (ab) in the table and figure. BIS = Behavioral Inhibition System scale; BAS_r = Behavioral Activation System scale_Reward responsiveness; BAS_d = Behavioral Activation System scale_Drive; BAS_f = Behavioral Activation System scale_Fun seeking; BDI = Beck Depression Inventory; BAI = Beck Anxiety Inventory; BIS-11 = Barratt Impulsiveness Scale; and STAXI = State-Trait Anger Expression Inventory.
